# The relationship between causes of suicidal attempts in Iran and individual and social variables: a retrospective study

**DOI:** 10.1186/s12888-022-04449-2

**Published:** 2022-12-12

**Authors:** Aliasghar Manouchehri, Zohreh Hosseini Marznaki, Letizia Maria Atim, Mehdi Mohammadian amiri, Mark Mohan Kaggwa

**Affiliations:** 1 Department of Internal Medicine, Shahid Beheshti Hospital Babol University of Medical Sciences, Babol, Iran; 2 Imam Ali Hospital at Amol City, Mazandaran University of Medical Sciences, Sari, Iran; 3 African Centre for Suicide Prevention and Research, Mbarara, Uganda; 4 Department of Psychiatry, Mbarara University of Science and Technology, Mbarara, Uganda; 5 Department of Emergency Medicine, School of Medicine, Babol University of Medical Sciences, Mazandaran, Iran; 6 Department of Psychiatry and Behavioural Sciences, McMaster University, Hamilton, Ontario Canada

**Keywords:** Suicidal attempts, Suicide, Causes, Romantic relationship problems, Substance use, Economic problems, Iran

## Abstract

**Objective:**

Determine the prevalence of suicide attempts and the relationships between the different causes of attempts with sociodemographic and clinical characteristics among individuals in Iran.

**Methods:**

A retrospective review of data about suicide attempts from poisoning care centers in Babol city between 2017 and 2021. Multinomial regression analysis (with mental illness being the reference variable) was used to determine the factors associated with the different causes of suicide attempts (addiction, romantic relationship problems, and economic problems).

**Results:**

The overall prevalence of completed suicide in the population sampled was 10.8% (95% confidence interval 9.5–12.1) (244/2,263). Relative to mental disorder, given that other variables in the model are held constant the following were associated with suicide attempts. A previous history of suicide attempts was associated with increasing the relative risk ratio of attempting suicide while having no positive history of smoking was associated with reducing the relative risk ratio of a suicidal attempt. However, the use of multiple drugs to attempt suicide was associated with an increased relative risk ratio of attempting suicide with romantic relationship problems and addiction as causes of suicide attempts. The first year of data collection (2017) and the female gender were both associated with an increased relative risk ratio of having a suicide attempt due to romantic relationships and economic problems. A family history of suicide was associated with an increased relative risk ratio of suicide attempts due to romantic relationship problems. However, using Pesticides-aluminum phosphide and detergent and javel water to attempt reduced the relative risk ratio of attempting due to romantic relationship problems. Age, self-employment, middle income, and married were associated with an increased relative risk ratio of suicide attempts among individuals due to addiction. However, staying longer at the emergency department was associated with a reduced relative risk ratio of having had a suicide attempt due to addiction.

**Conclusions:**

This study highlights the interplay between romantic hardships, addiction, economic hardships as reasons for suicide attempts and various sociable variables in a population in Northern Iran. The most associated reason for suicide attempts was romantic relationship hardships. Therefore, interventions such as sessions on conflict resolution, boundary setting, and management of grieving would greatly benefit this society and reduce the rate of suicide, especially among individuals with a history of suicide attempts.

**Supplementary Information:**

The online version contains supplementary material available at 10.1186/s12888-022-04449-2.

## Introduction

The recent suicide rate (2019) in Iran is 5.2 per 100,000 people [1], a decrease from 7.91 per 100,000 people (in 2014) [2]. However, the rate of suicide attempts was extremely high (i.e., 193.49 per 100,000) [2], and no recent study has determined the current rate. Among older persons in Iran, the rate of suicide attempts and suicide were 21.47 and 4.52 per 100,000 population between 2011 and 2016, respectively [3]. The suicide attempts and suicide rates in older persons (in the same period) were lower than that among adolescents, 193.49 and 7.91 per 100,000, respectively [4]. This indicates an increase in suicidal behaviors among the young generation. No wonder, in 2020, Iran recorded over 1.5 million suicide cases, the highest in most Islamic countries [5]. The high rate of suicide and suicide attempts may be associated with the previously identified factors, such as (i) sociodemographic (i.e., gender, age, marital status, economic status, education status. area of residence, sociocultural status), (ii) Social factors (i.e., family conflicts/problems, financial problems, unemployment, romantic relationship problems), (iii) biological (i.e., medication side effects, use of psychoactive substances, physical illnesses, genetics/family history of suicidal behaviors), and (iv) psychological (i.e., trauma, psychological distress, mental illness) [4, 6–19]. However, many studies have demonstrated the interconnectedness between the various risk factors and their association with suicidal attempts [20]. Various studies about suicide and suicidal behaviors in Iran have reported several factors/causes associated with suicide attempts or suicide [3, 4, 9, 21]. However, no detailed emphasis or analysis has focused on exploring the relationship/associations related to the commonly identified causes. Identifying such relationships can enable clinicians and policymakers to find solutions to mitigate or reduce the impact of various causes by finding solutions to the common associated factors among the various causes.

Individuals admitted to hospital emergencies have consistently been reported to have the following prevalent reasons for the causes of attempts, i.e., romantic relationship problems, addiction, economic problems, and mental disorders [6–8]. This study, therefore, seeks to understand the factors associated with the different causes of suicide attempts. Identifying this information will assist healthcare workers, policymakers, and caregivers of individuals at risk in watching out or planning for strategies to combat suicide attempts.

## Methods

In this retrospective study, we captured data regarding suicide attempts from the poisoning care centers in Babol city in north of iran. This study obtained approval from the Ethics Committee of the Babol University of Medical Science (Approval#: IR.MUBABOL.HRI.REC.1401.002).

### Data collection and management

The poisoning care centers have questionnaires that collect demographic information, past medical history, history of suicide, and data on the suicide cause, method of suicide, and outcome. We designed an online data caption tool that captured the above information. Two independent research assistants entered data, and the first author resolved any discrepancies such as. The final excel sheet was compiled by the first author and who also performed data cleaning.

### Data analysis

Data was exported to SPSS Version 25 software for data analysis. Percentages and frequencies summarized the demographics and clinical characteristics for categorical variables. However, mean, standard deviation, minimum, and maximum values were presented for continuous variables. Normality was tested using Kolmogorov Smirnov. The Chi-square test was used to test for statistical differences between the different causes of suicidal attempts and the categorical study variables. While ANOVA was used for the continuous variables. Multinomial regression analysis (with mental illness being the reference variable) was used to determine the factors associated with the different causes (addiction, romantic relationships, and economic problems) of suicide attempts among residents of Babol city in north of iran. A *p*-value < 0.05 was set to be statistically significant.

## Results

### Participants’ characteristics

About 73.1% of (2263) cases who went to the hospital due to suicide attempts were women. The mean age was 45.53 ± 13.35 years. The average length of stay at the emergency department was 2.78 ± 1.71 days. Most cases (50.5%) did not have insurance, and 59.9% resided in rural areas. Many of the referred adolescents were high schoolers (37.3%) and self-employed (37.5%), and unemployed (32.9%). Romantic relationship problems were responsible for most suicide attempts (41.7%). The overall prevalence of completed suicide in the population sampled was 10.8% (95% CI 9.5 – 12.1) (Table 1).

**Table 1:** Description of demographic and clinical variables in the research population

Variable	Category	N (%)	Variable	Category	N (%)
**Gender**	Female	608(26.9)	**Cause of suicide**	Romantic relationship problems	943(41.7)
	Male	1655(73.1)		Addiction	354(15.6)
**Insurance**	Not insured	1142(50.5)		Economic problems	530(23.4)
	Iran Health Insurance Organization	422(18.6)		Mental disorders	436(19.3)
	Social Security Organization	403(17.8)	**Survived or dead**	Survived	2019(89.2)
	General health insurance	224(9.9)		Dead	244(10.8)
	Others	72(3.2)	**Poison drug used**	Opium	361(16.0)
**Marriage status**	Married	772(34.1)		Pesticides	385(17.0)
	Single	831(36.7)		Amphetamine	123(5.4)
	Widowed-Divorced	660(29.2)		Detergent	243(10.7)
**Resident**	Rural	1356(59.9)		Antiepileptic’s	587(25.9)
	Urban	907(40.1)		Alcohol	216(9.5)
**Income**	Low	1124(49.7)		Acetaminophen	118(5.2)
	Middle	801(35.4)		TCA	163(7.2)
	High	338(14.9)		Multiple drug	10(.4)
**Smoking**	No	879(38.8)		Bـ blocker	57(2.5)
	Yes	1384(61.2)	**GCS**	3–8 (low)	815(36.0)
**Alcohol**	No	2242(99.1)		9–12 (medium)	893(39.5)
	Yes	21(.9)		13–15 (high)	555(24.5)
**Season suicide**	Spring	440(19.4)	**History of suicide attempt in case**	No	992(43.8)
	Summer	351(15.5)		Yes	1271(56.2)
	Autumn	1045(46.2)	**Family history of suicide**	No	1845(81.5)
	Winter	427(18.9)		Yes	418(18.5)
**Education**	Primary school and less	837(37.0)	**History of at least one suicide attempt**	No	2094(92.5)
	High school	844(37.3)		Yes	169(7.5)
	Diploma and graduate diploma	455(20.1)	**Physical illness**	No	1980(87.5)
	Bsc-msc-Doctorate	127(5.6)		Yes	283(12.5)
**Job**	Self-employment	849(37.5)			
	Employee	609(26.9)			
	Unemployed	744(32.9)			
	Retired	61(2.7)			
**Year**	2017	560(24.7)			
	2018	532(23.5)			
	2019	338(14.9)			
	2020	571(25.2)			
	2021	262(11.6)			

### Relationship between sociodemographic variables and causes of suicide attempt

Based on the different causes, males statistically attempted suicide more than females, with most of the causes being an addiction, followed by economic problems, then mental disorders, and then romantic relationship problems (80.2%, 74.5%, 71.8%, and 70.3%, respectively; *p* = 0.003). Most of the suicide attempts were among individuals who were not insured, and romantic relationship problems caused more attempts than other causes (54.0%, 50.0%, 48.0%, and 46.2%. for romantic relationship problems, mental disorders, addiction, and economic problems, respectively; *p* < 0.001). Most attempts were among single individuals, and romantic relationship problems statistically caused more attempts than other causes (40.5%, 39.5%, 33.0%, and 31.1%, for romantic relationship problems, addiction, mental disorders, and economic problems causes, respectively; *p* < 0.001). Urban residents attempted suicide mainly due to addiction (55.4%), while rural residents mostly attempted suicide due to romantic relationship problems (63.6%) (*p* < 0.001). Cases with low income mostly attempted due to mental disorders (55.3%) and romantic relationship problems (52.3%). While cases with a high income are mostly because of romantic relationship problems (17.6%) and economic problems (15.5%); (*p* < 0.001). Most of the individuals who smoked attempted suicide, and the leading cause was due to addiction as compared to other causes (66.9%, 63.4%, 59.5%, and 57.3%, for addiction, economic problems, romantic relationship problems, and mental disorders causes, respectively, *p* = 0.019). Most suicide attempts were among individuals whose education was primary level or below, and most of them attempted suicide due to romantic relationship problems compared to other causes (45.3%, 38.3%, 27.7%, and 27.4%, for romantic relationship problems, addiction, and economic problems, respectively; *p* < 0.001). Significantly, most individuals who were self-employed attempted due to economic problems (43.0%), most employees due to addiction (35.0%), unemployed due to romantic relationship problems (36.5%), and retired due to mental disorders (5.5%). Most of the cases who had a history of suicide in the case attempted suicide due to romantic relationship problems (59.2%) and mental disorders (57.6%) (*p* = 0.024). Most individuals with a history of at least one attempted suicide attempted due to Mental disorders (24.8%) and mental disorders (57.6%) (*p* < 0.001). Most of the cases who had a physical illness attempted suicide due to romantic relationship problems (14.7%) and mental disorders (13.1%) (*p* = 0.016). In the first year of the study, fewer individuals attempted suicide due to addiction (11.6%), while in the last year (fifth) of the study, 17.2% attempted for this reason (*p* < 0.001) (Table 2).

**Table 2:** Comparison the frequency of demographic variables by the Cause of suicide in the research population

Variable	Category	Romantic relationship problems	Addiction	Economic problems	Mental disorders	Statist (df) *P*-value
**Sex**	Female	280(29.7)	70(19.8)	135(25.5)	123(28.2)	13.82 (3) 0.003
	Male	663(70.3)	284(80.2)	395(74.5)	313(71.8)	
**Insurance**	Not insured	509(54.0)	170(48.0)	245(46.2)	218(50.0)	44.28 (12) < 0.001
	Iran health insurance organization	166(17.6)	49(13.8)	124(23.4)	83(19.0)	
	Social security organization	160(17.0)	89(25.1)	72(13.6)	82(18.8)	
	General health insurance	88(9.3)	31(8.8)	65(12.3)	40(9.2)	
	Others	20(2.1)	15(4.2)	24(4.5)	13(3.0)	
**Marriage status**	Married	251(26.6)	137(38.7)	221(41.7)	163(37.4)	50.48 (6) < 0.001
	Single	382(40.5)	140(39.5)	165(31.1)	144(33.0)	
	Widowed-divorced	310(32.9)	77(21.8)	144(27.2)	129(29.6)	
**Resident**	Rural	600(63.6)	157(44.4)	324(61.1)	275(63.1)	43.26 (3) < 0.001
	Urban	343(36.4)	197(55.6)	206(38.9)	206(36.9)	
**Income**	Low	493(52.3)	129(36.4)	261(49.2)	241(55.3)	72.28 (6) < 0.001
	Middle	284(30.1)	192(54.2)	187(35.3)	138(31.7)	
	High	166(17.6)	33(9.3)	82(15.5)	57(13.1)	
**Smoking**	No	382(40.5)	117(33.1)	194(36.6)	186(42.7)	9.90 (3) 0.019
	Yes	561(59.5)	237(66.9)	336(63.4)	250(57.3)	
**Alcohol**	No	938(99.5)	347(98.0)	525(99.1)	432(99.1)	5.87 (3) 0.118
	Yes	5(0.5)	7(2.0)	5(0.9)	4(0.9)	
**Season**	Spring	194(20.6)	58(16.4)	102(19.2)	86(19.7)	11.47 (9) 0.245
	Summer	158(16.8)	53(15.0)	71(13.4)	69(15.8)	
	Autumn	420(44.5)	177(50.0)	262(49.4)	186(42.7)	
	Winter	171(18.1)	66(18.6)	95(17.9)	95(21.8)	
**Education**	Primary school and less	427(45.3)	98(27.7)	145(27.4)	167(38.3)	72.28 (6) < 0.001
	High school	306(32.4)	164(46.3)	221(46.3)	153(35.1)	
	Diploma and graduate diploma	172(18.2)	73(20.6)	127(24.0)	83(19.0)	
	Bsc-msc-doctorate	38(4.0)	19(5.4)	37(7.0)	33(7.6)	
**Job**	Self-employment	337(35.7)	130(36.7)	228(43.0)	154(35.3)	72.28 (6) < 0.001
	Employee	241(25.6)	124(35.0)	131(24.7)	113(25.9)	
	Unemployed	344(36.5)	96(27.1)	159(30.0)	145(33.3)	
	Retired	21(2.2)	4(1.1)	12(2.3)	24(5.5)	
**History of suicide in case**	No	385(40.8)	172(48.6)	250(47.2)	185(42.4)	9.46 (3) 0.024
	Yes	558(59.2)	182(51.4)	280(52.8)	251(57.6)	
**Family history of suicide**	No	777(82.4)	284(80.2)	433(81.7)	351(80.5)	1.18 (3) 0.757
	Yes	166(17.6)	70(19.8)	97(18.3)	85(19.5)	
**History of at least one**	No	908(96.3)	346(97.7)	512(96.6)	328(75.2)	234.76 (3) < 0.001
	Yes	35(3.7)	8(2.3)	18(3.4)	108(24.8)	
**Physical illness**	No	804(85.3)	317(89.5)	480(90.6)	379(86.9)	10.37 (3) 0.016
	Yes	139(14.7)	37(10.5)	50(9.4)	57(13.1)	
**Year**	2017	347(36.8)	41(11.6)	115(21.7)	57(13.1)	212.65 (12) < 0.001
	2018	173(18.3)	55(15.5)	168(31.7)	136(31.2)	
	2019	142(15.1)	72(20.3)	60(11.3)	64(14.7)	
	2020	175(18.6)	125(53.3)	138(26.0)	133(30.5)	
	2021	106(11.2)	61(17.2)	49(9.2)	46(10.6)	

### Relationship between clinical variables and causes of suicide attempt

Most of the individuals who attempted suicide survived, and the least individuals who died after an attempt it was related to mental disorders (5.0%). Most of the individuals who attempted due to addiction used methadone-opium-tramadol (45.2%) as the poison drug, while those due to economic problems used pesticides-aluminum phosphide (29.8%) (*p* < 0.001). Most cases with a low level of consciousness had addiction (42.4%) and economic problems (43.4%) as the reason for attempting suicide. In comparison, most of the cases who had a high level of consciousness had mental disorders (60.6%) (*p* < 0.001) (Table 3).

**Table 3:** Comparison the frequency of clinical variables by Cause of suicide in the research population

Variable	Category	Romantic relationship problems	Addiction	Economic problems	Mental disorders	Statist (df) *P*-value
					Frequency (%)	
Survived or dead	Survived	831(88.1)	309(87.3)	465(87.7)	414(95.0)	18.67 (3) < 0.001
	Dead	112(11.9)	45(12.7)	65(12.3)	22(5.0)	
Poison drug used	Methadone-opium-tramadol	114(12.1)	160(45.2)	60(11.3)	27(6.2)	1727.57 (27) < 0.001
	Pesticides-alminium phosphid	94(10.0)	39(11.0)	158(29.8)	94(21.6)	
	Hashish-amphetamin	37(3.9)	32(9.0)	47(8.9)	7(1.6)	
	Detergent and javel water	8(0.8)	0(0.0)	0(0.0)	235(53.9)	
	Antiepileptic’s	414(43.9)	59(16.7)	80(15.1)	34(7.8)	
	Alcohol	154(16.3)	17(4.8)	30(5.7)	15(3.4)	
	Acetaminophen	23(2.4)	16(4.5)	72(13.6)	7(1.6)	
	TCA	60(6.4)	29(8.2)	61(11.5)	13(3.0)	
	Muity drug	7(0.7)	1(0.3)	2(0.4)	0(0.0)	
	B blocker	32(3.4)	1(0.3)	20(3.8)	4(0.9)	
**GCS**	Low	347(36.8)	150(42.4)	230(43.4)	88(20.2)	392.74 (6) < 0.001
	Medium	454(48.1)	139(39.3)	216(40.8)	84(19.3)	
	High	142(15.1)	65(18.4)	84(15.8)	264(60.6)	

### Relationship between continuous variables and causes of suicide attempt

The average age of cases who died because of romantic relationship problems (44.57 ± 15.11) is lower than in other cases (*p* = 0.037). Addiction has the lowest average (2.28 ± 1.43) length of stay at the emergency department, and economic problems have the highest (3.00 ± 2.05) average (*p* < 0.001). Romantic relationship problems have the lowest (13.68 ± 6.66) average time, and addiction has the highest (14.65 ± 6.51) average (*p* = 0.037) (Table 4).

**Table 4:** Comparison the Mean of quantitative variables by the Cause of suicide in the research population

Variable	Category	N	Mean ± SD	Statist (df) *P*-value
**Age**	Romantic relationship problems	943	44.57 ± 15.11	2.82 (3) 0.037
	Addiction	354	46.37 ± 10.67	
	Economic problems	530	46.23 ± 11.85	
	Mental disorders	436	46.08 ± 12.85	
**Length stay at emergency department**	Romantic relationship problems	943	2.83 ± 1.59	13.43 (3) < 0.001
	Addiction	354	2.28 ± 1.43	
	Economic problems	530	3.00 ± 2.05	
	Mental disorders	436	2.79 ± 1.67	
**time**	Romantic relationship problems	943	13.68 ± 6.66	2.47 (3) 0.037
	Addiction	354	14.65 ± 6.51	
	Economic problems	530	13.83 ± 6.64	
	Mental disorders	436	14.36 ± 6.41	

### Factors associated with causes of suicide attempt

Table 5 shows all study variables with the different causes of suicide by multinomial regression and mental disorders considered as a reference. Given the other variables in the model are kept constant, the following factors were associated with an increase in the relative risk ratio of attempting suicide in all the different causes relative to mental disorders: history of suicide attempt and history of at least one previous attempt, having no positive. History of smoking was associated with a reduction in the relative risk ratio of attempting suicide in all the different causes relative to mental disorders, given the other variables in the model are kept constant.

**Table 5:** Investigating of all variables with the Causes of suicide by multinomial regression

		**Cause of suicide**					
		**Romantic relationship problems**		**Addiction**		**Economic problems**	
		**RRR**	**Wald** **(*p*-value)**	**RRR**	**Wald** **(*p*-value)**	**RRR**	**Wald** **(*p*-value)**
**Age**		0.99(.01)	.54 (.464)	1.04(.01)	9.912 (.002)	1.01(.01)	.96 (.33)
**Length stay at emergency department**		1.03(.06)	.19 (.663)	.79(.08)	8.71 (.003)	1.01(.06)	.02 (.86)
**Time**		1.01(.01)	.01 (.965)	1.01(.02)	.40 (.524)	1.00(.02)	.02 (.87)
**Sex**	**Female**	2.90(.33)	10.70 (.001)	1.98(.37)	3.34 (.068)	2.51(.34)	7.17 (.007)
	**Male**						
**Resident**	**Rural**	1.15(.22)	.41 (.522)	.99(.25)	.01 (.959)	1.19(.23)	.58 (.446)
	**Urban**						
**Education**	**Primary school and less**	1.51(.51)	.65 (.420)	1.06(.58)	.01 (.915)	.80(.52)	.19 (.660)
	**High school**	1.13(.49)	.06 (.802)	1.57(.55)	.67 (.414)	.94(.50)	.01 (.903)
	**Diploma and graduate diploma**	1.26(.50)	.22 (.641)	1.41(.55)	.39 (.534)	1.28(.50)	.24 (.620)
	**Bsc-msc-Doctorate**						
**Job**	**Self-employment**	2.04(.58)	1.48 (.224)	5.43(.77)	4.78 (.029)	2.66(.62)	2.49 (.114)
	**Employee**	1.46(.60)	.41 (.524)	4.44(.78)	3.63 (.057)	1.79(.63)	.85 (.356)
	**Unemployed**	1.42(.59)	.34 (.559)	2.79(.78)	1.72 (.190)	1.49(.63)	.39 (.529)
	**Retired**						
**Income**	**Low**	.56(.33)	3.25 (.072)	1.14(.39)	.11 (.740)	.580(.34)	2.62 (.105)
	**Middle**	.58(.34)	2.56 (.110)	2.89(.40)	7.13 (.008)	.99(.35)	.01 (.967)
	**High**						
**Marriage status**	**Married**	.81(.27)	.65 (.422)	2.70(.31)	10.18 (.001)	1.46(.28)	1.84 (.175)
	**Single**	.93(.27)	.08 (.784)	1.73(.32)	2.98 (.084)	1.12(.28)	.16 (.690)
	**Widowed-Divorced**						
**Insurance**	**Not insured**	1.59(.65)	.51 (.474)	.53(.69)	.82 (.367)	.83(.65)	.08 (.769)
	**Iran Health Insurance Organization**	2.23(.67)	1.45 (.229)	.83(.71)	.07 (.794)	1.71(.66)	.66 (.415)
	**Social Security Organization**	1.80(.67)	.76 (.383)	1.38(.71)	.20 (.652)	.87(.67)	.04 (.834)
	**General health insurance**	2.65(.71)	1.88 (.171)	.55(.76)	.64 (.426)	1.36(.70)	.19 (.663)
	**Others**						
**Year**	**2017**	4.36(.37)	15.90 (.001)	.49(.43)	2.80 (.094)	2.74(.39)	6.62 (.010)
	**2018**	1.56(.40)	1.28 (.260)	.56(.45)	1.65 (.199)	1.50(.41)	.97 (.324)
	**2019**	1.31(.37)	.54 (.462)	.87(.41)	.11 (.740)	1.10(.40)	.06 (.804)
	**2020**	.70(.36)	.97 (.325)	1.37(.40)	.63 (.426)	.90(.39)	.08 (.782)
	**2021**						
**Season**	**Spring**	1.31(.30)	.82 (.364)	.95(.34)	.02 (.880)	1.27(.31)	.59 (.441)
	**Summer**	1.16(.31)	.22 (.639)	.93(.36)	.04 (.834)	.99(.33)	.01 (.975)
	**Autumn**	1.35(.25)	1.42 (.234)	1.49(.29)	1.91 (.167)	1.60(.26)	3.16 (.075)
	**Winter**						
**SMOKING**	**No**	.53(.28)	5.10 (.024)	.53(.31)	4.18 (.041)	.49(.29)	5.86 (.015)
	**Yes**						
**ALCHOL**	**No**	.33(1.19)	.86 (.354)	.10(1.24)	3.60 (.058)	.48(1.24)	.35 (.551)
	**Yes**						
**History of suicide in case**	**No**	1.75(.21)	7.08 (.008)	1.74(.24)	5.40 (.020)	2.32(.22)	14.83 (.001)
	**Yes**						
**Family history of suicide**	**No**	1.99(.25)	7.36 (.007)	1.24(.29)	.55 (.459)	1.61(.27)	3.19 (.074)
	**Yes**						
**History of at least one**	**No**	33.19(.28)	156.43 (.001)	40.08(.43)	73.45 (.001)	34.98(.32)	125.75 (.001)
	**Yes**						
**Physical illness**	**No**	.96(.29)	.02 (.876)	1.28(.33)	.55 (.457)	1.25(.30)	.53 (.464)
	**Yes**						
**GCS**	**Low**	2.20(.33)	5.69 (.017)	1.43(.37)	.91 (.340)	1.93(.34)	3.67 (.055)
	**Medium**	1.81(.28)	4.45 (.035)	1.24(.32)	.47 (.493)	1.61(.29)	2.64 (.104)
	**High**						
**Poison drug**	**Methadone-opium-tramadol**	.57(.69)	.68 (.409)	8.85(1.22)	3.19 (.074)	.34(.71)	2.28 (.131)
	**Pesticides-alminium phosphid**	.14(.66)	8.65 (.003)	.57(1.21)	.22 (.642)	.29(.67)	3.34 (.068)
	**Hashish-amphetamin**	.92(.80)	.01 (.918)	10.27(1.29)	3.27 (.070)	1.49(.81)	.24 (.624)
	**Detergent and javel water**	.01(.75)	52.25 (.001)	0.01(5868.85)	.00 (.997)	0.01(5126.11)	.00 (.996)
	**Antiepileptic’s**	2.68(.68)	2.12 (.146)	3.28(1.22)	.95 (.329)	.61(.700)	.48 (.487)
	**Alcohol**	1.30(.71)	.14 (.713)	2.27(1.26)	.43 (.514)	.37(.75)	1.73 (.189)
	**Acetaminophen**	.99(.79)	.00 (.997)	8.20(1.29)	2.66 (.103)	4.26(.78)	3.43 (.064)
	**Tca**	.47(.72)	1.11 (.293)	3.13(1.25)	.84 (.360)	.79(.74)	.11 (.744)
	**Muity drug**	41,421,970.78(.89)	391.56 (.001)	232,756,254.50(1.71)	126.99 (.001)	28,543,623.39(.01)	
	**B blocker**						
**Survived or dead**	**Survived**	.43(.36)	5.59 (.018)	.55(.39)	2.28 (.131)	.87(.37)	.15 (.691)
	**Dead**						

However, the use of multiple drugs to attempt suicide was associated with an increased relative risk ratio of attempting suicide in both romantic relationship problems and addiction relative to mental disorder, given other variables in the model are held constant.

The first year of data collection (2017) and the female gender were both associated with an increased relative risk ratio of suicidal attempt with romantic relationships and economic problems relative to mental disorder, given other variables in the model are held constant.

Family history of suicide increased the relative risk ratio of suicide attempt among individuals with romantic relationship problems, relative to mental disorder, given other variables in the model are held constant. However, using Pesticides-aluminum phosphide and detergent and javel water to attempt reduced the relative risk ratio of attempting among those with romantic relationship problems relative to mental disorder, given other variables in the model are held constant.

Age, self-employment, middle income, and married were associated with an increased relative risk ratio of suicide attempt among individuals having an addiction relative to mental disorder, given other variables in the model are held constant. However, staying longer at the emergency department was associated with a reduced relative risk ratio of having had a suicide attempt due to addiction relative to mental disorder, given other variables in the model are held constant.

## Discussion

In this cross-sectional study in Babol city of Northern Iran, we aimed to determine the factors associated with the different reasons for attempting suicide (addiction, romantic relationships, and economic problems) relative to mental disorders. The overall prevalence of completed suicide in the population sampled was 10.9% (95% CI 9.5 – 12.1). The prevalence is similar to that reported elsewhere in Iran, particularly in Najafabad, in the center, perhaps due to similarity in culture and social variables influencing suicidal behavior [9]. Similar to suicide rates, the number of suicide attempts per 100,000 individuals is lower than previous estimates (i.e., 193.49/100,000) [2]. This indicates the country's progressively important suicide interventional and preventative programs, such as the Iran National suicide prevention Program [21]. However, the prevalence is slightly higher than the 5% reported in Uganda [8]. Notably, however, this information in that study was derived through medical records, which could have been incomplete, hence estimating a lower prevalence. Despite the efforts by the different programs, more efforts are needed to combat suicidal behaviors in Iran. This may involve both government and nongovernment programs. Newer interventions, such as using pets, can reduce suicidal behaviors, as seen in other parts of the world [22]. Surprisingly, unlike most studies the rate of suicide attempts was higher among men than women [23, 24] due to higher prevalence of major depression among women than men. This may suggest a shift in mental illness epidemiology.

The relative risk ratio for suicide attempts due to all studied causes increased among individuals with previous suicide attempts relative to mental disorder, given other variables in the model are held constant. This is because they tend to continue having ongoing suicidal ideations with worse lethality and therefore try whatever methods possible to end their lives [25]. Furthermore, due to the shame and stigma they face after the first attempt, many find life unbearable and therefore try to end it by all means [26, 27]. In addition, individuals without mental illness who were smokers attempted suicide due to romantic relationships, addiction, and economic problems. Having no positive history of smoking reduced the relative risk ratio of suicide attempt in the present study relative to mental disorder, given other variables in the model are held constant, a non-surprising finding based on previous literature. Cigarette smoking is a risk factor for developing chronic illnesses such as cancer, hypertension, and heart disease; these illnesses may predispose one to depression and hence suicide. Not to mention that smoking may also alter brain chemistry leading to depression, feeling of hopelessness, and suicide [28]. However, smoking is associated with suicidality without the intermediary of depression [29]. This could be because nicotine activates the responsiveness of the Hypothalamus–Pituitary–Adrenal axis to psychological stress [30].

Among those without mental illness, those who attempted suicide using multiple drugs had an addiction and economic problems. Those with addiction tend to spend all their money buying substances, face economic problems, fail to contribute to their family needs and feel life is not worth living [31]. Thus, higher motives to end their lives and end up using multiple drugs. This situation is similar to those with economic hardship, which, after failing to be financially happy, try by all means to end their lives and believe multiple drugs may be the best option.

Females without mental illness attempted suicide due to romance and economic problems. Notwithstanding, those who attempted suicide by using pesticides and detergents did so due to romantic relationship problems. Due to the distress from romantic relationships, women attempt suicide to control their significant other or seek attention without a solid intention to die as opposed to men [18, 32–34]. Therefore, they attempted suicide by less lethal means, such as using pesticides and detergents. Furthermore, economic problems create social vulnerabilities among women, which may make a life for females unbearable, causing them to want to commit suicide [35].

Participants with a family history of suicide without mental illness attempted suicide due to romantic relationships. Suicidal behavior has been linked to over 300 genes in addition to environmental factors [36]. Family members do not only share a genetic susceptibility but similar living conditions, social values, and ways of conflict resolution. Relationship dynamics, therefore, can be stressful events triggering suicidal ideas in people at risk [37, 38].

In participants without mental illness, with increasing age, tended to attempt suicide due to addiction problems. This is no wonder because, with increasing age, functional decline sets in, individuals are also more exposed to addictive substances, and substance use disorders worsen [39]. They also utilize all their resources in acquiring these substances and feel useless to their society hence suicidal ideas and suicide attempts. Furthermore, acute and chronic utilization of substances may impair judgment culminating in impulsive behavior such as suicidal acts [40]. Those who were self-employed also attempted suicide due to addiction problems. This could be because they are more economically empowered and are able to obtain the drugs of addiction. Addiction problems were also the reason for attempting suicide among married participants without mental illness. Addiction problems are major problems for marital discord, verbal and physical aggression, financial difficulties, and sometimes divorce [41]. This culminates in feelings of extreme stress, worthlessness, and in some cases, eventually suicidal ideations.

Individuals without mental illness who attempted suicide due to addiction problems also stayed in the emergency for a short time. Emergency care is short-term and meant to manage crises. However, suicide attempters with addiction problems usually require long-term management and, in another setting, other than the emergency room to fully work their disorder. This could explain why they spent a shorter time in the emergency.

The was a statistical difference between the average age of individuals having different causes for suicide attempt, with individuals’ relationship problems being younger than other groups. Relationship problems are common causes for suicidal behaviors and suicide among young individuals[15, 18, 19]. However, these average ages were above the common national age group for individuals who attempt or die by suicide (15 – 24 years) [9, 42]. The other age group that has been reported to have high level of suicide attempt or dying by suicide in Iran are those above 65 years [3, 42], this could have lead to the average age of individuals with suicidal attempts to be between 44 and 46 years.

### Strength and limitations

The major strength of this study is that it is a retrospective study with a large sample size from a poisoning center in the Northern part of Iran, a region currently with diverse culture representative of most of Iran, thus, making the findings generalizable to other parts of the country. However, the study has a few limitations. The study had numerous variables having missing data on different that could have different finding in the multi regression model. However, the sample was large to compensate for such small differences. Another limitation to the study finding and their interpretation is that the study was based on reports from patients or their relatives about the cause of suicide attempt/suicide. This subjective reporting is prone to giving socially desirable findings/responses since there is a lot of stigma towards suicide in Iran [43].

## Conclusions

This study highlights the interplay between romantic hardships, addiction, and economic hardships as reasons for suicide attempts and various sociable variables in a population in Northern Iran. The most associated reason for suicide attempts is romantic hardships. Interventions such as sessions on conflict resolution, boundary setting, and management of grieving would greatly benefit this society and reduce the rate of suicide, especially among individuals with a history of suicide attempts.

## Supplementary Information

**Additional file 1. **

## Data Availability

All data used in this manuscript are provided as a supplementary document.
